# The relationship between major depression and migraine: A bidirectional two-sample Mendelian randomization study

**DOI:** 10.3389/fneur.2023.1143060

**Published:** 2023-04-14

**Authors:** Xiaofeng Lv, Bojun Xu, Xiurong Tang, Shanshan Liu, Jun-Hui Qian, Julan Guo, Jian Luo

**Affiliations:** ^1^Hospital of Chengdu University of Traditional Chinese Medicine, Chengdu, Sichuan, China; ^2^School of Acupuncture and Tuina, Chengdu University of Traditional Chinese Medicine, Chengdu, China; ^3^Guang’an Traditional Chinese Medicine Hospital, Guang’an, Sichuan, China

**Keywords:** Mendelian randomization, major depressive disorder, migraine, genome-wide association study, causal association, bidirectional

## Abstract

**Background:**

Previous epidemiological and other studies have shown an association between major depressive disorder (MDD) and migraine. However, the causal relationship between them remains unclear. Therefore, this study aimed to investigate the causal relationship between MDD and migraine using a bidirectional, two-sample Mendelian randomization (MR) approach.

**Methods:**

Data on MDD and migraine, including subtypes with aura migraine (MA) and without aura migraine (MO), were gathered from a publicly available genome-wide association study (GWAS). Single nucleotide polymorphisms (SNPs) utilized as instrumental variables (IVs) were then screened by adjusting the intensity of the connection and removing linkage disequilibrium. To explore causal effects, inverse variance weighting (IVW) was used as the primary analysis method, with weighted median, MR-Egger, simple mode, and weighted mode used as supplementary analytic methods. Furthermore, heterogeneity and pleiotropy tests were carried out. Cochran’s Q-test with IVW and MR-Egger was used to assess heterogeneity. Pleiotropy testing was carried out using the MR-Egger intercept and MR-PRESSO analysis methods. A leave-one-out analysis was also used to evaluate the stability of the findings. Finally, we used migraine (MA and MO) levels to deduce reverse causality with MDD risk.

**Results:**

Random effects IVW results were (MDD-Migraine: odds ratio (OR), 1.606, 95% confidence interval (CI), 1.324–1.949, *p* = 1.52E-06; MDD-MA: OR, 1.400, 95%CI, 1.067–1.8378, *p* = 0.015; MDD-MO: OR, 1.814, 95%CI, 1.277–2.578, *p* = 0.0008), indicating a causal relationship between MDD levels and increased risk of migraine (including MA and MO). In the inverse MR analysis, the findings were all negative, while in sensitivity analyses, the results were robust except for the study of MA with MDD.

**Conclusion:**

Our study confirms a causal relationship between MDD levels and increased risk of migraine, MA, and MO. There was little evidence in the reverse MR analysis to suggest a causal genetic relationship between migraine (MA and MO) and MDD risk levels.

## 1. Introduction

Migraine is the most prevalent disabling neurological disease, impacting more than 15% of the world’s population (1). It is characterized by recurrent headaches, often accompanied by nausea, vomiting, photophobia, and phonophobia (2). According to the Global Burden of Disease (GBD) survey, migraine negatively affects more than 6% of the population regarding earning capacity or employment (3). In addition, migraine was related to a 19.1% probability of anxiety, a 6.9% probability of depression, and a 5.1% probability of both (4).

With a worldwide prevalence of more than 300 million, major depressive disorder (MDD) is a prevalent psychiatric condition and is one of the primary sources of disability (5). The lifetime prevalence of MDD in adults in the United States is estimated at 17% (6). The economic cost of depression in the United States was $210 billion in 2010 (7). The World Health Organization predicts that MDD will be the leading cause of the global burden of disease by 2030 (8). MDD is a risk factor for multiple diseases and is emerging as a hot spot of concern for migraine progression (9, 10). A recent cross-sectional study found that MDD was strongly associated with the frequency and severity of attacks of migraine with aura, a subtype of migraine (11). Furthermore, in genetics, the importance of shared genetic factors between migraine and depression has been acknowledged (12). Although MDD and migraine are frequently seen together, their connection has not been thoroughly established. Therefore, whether there is a causal involvement in the onset of MDD and migraine is unknown.

Based on current research evidence, there are no randomized controlled trials on MDD and migraine for the time being; they are more observational and suffer from shortcomings such as reverse causality, small sample size, and confounding factors. The Mendelian randomization (MR) analysis is similar to randomized controlled trials (13), which use single nucleotide polymorphisms (SNPs) as instrumental variables (IVs) to establish a causative connection between exposure and outcome (14). Because alleles segregate randomly during meiosis without interference from external factors and genetic variants arise before disease, MR lowers bias induced by confounding factors and prevents interference from reverse causality (15, 16). This method has also been used more often in various clinical causal inferences. For example, Chen et al. (17) revealed a potential protective effect of intestinal flora on the pathogenesis of MDD by the MR analysis method. In addition, Yin et al. (18) demonstrated that lifelong elevated serum calcium increased the risk of migraine. More recent MR studies have analyzed separately the causal relationship between depression (19, 20) and migraine (21, 22) and other disorders. However, no studies have used MR methods to investigate the causal relationship between MDD and migraine. However, studies focusing on the association between MDD and migraine by an MR analysis are unavailable. Pisanu et al. (11) discovered that MDD subtypes were significantly associated with migraine subtypes, prevalence, and severity.

Therefore, migraine and migraine subtypes [migraine with aura (MA) and migraine without aura (MO)] were used for analysis in our study. We used pooled data from a large genome-wide association study (GWAS) using a bidirectional two-sample MR analysis to explain the common causal effect of MDD with the risk of migraine and its subtypes (MA and MO). The research may lay the groundwork for clinical treatment and prevention.

## 2. Materials and methods

### 2.1. Study design

**Figure 1:**
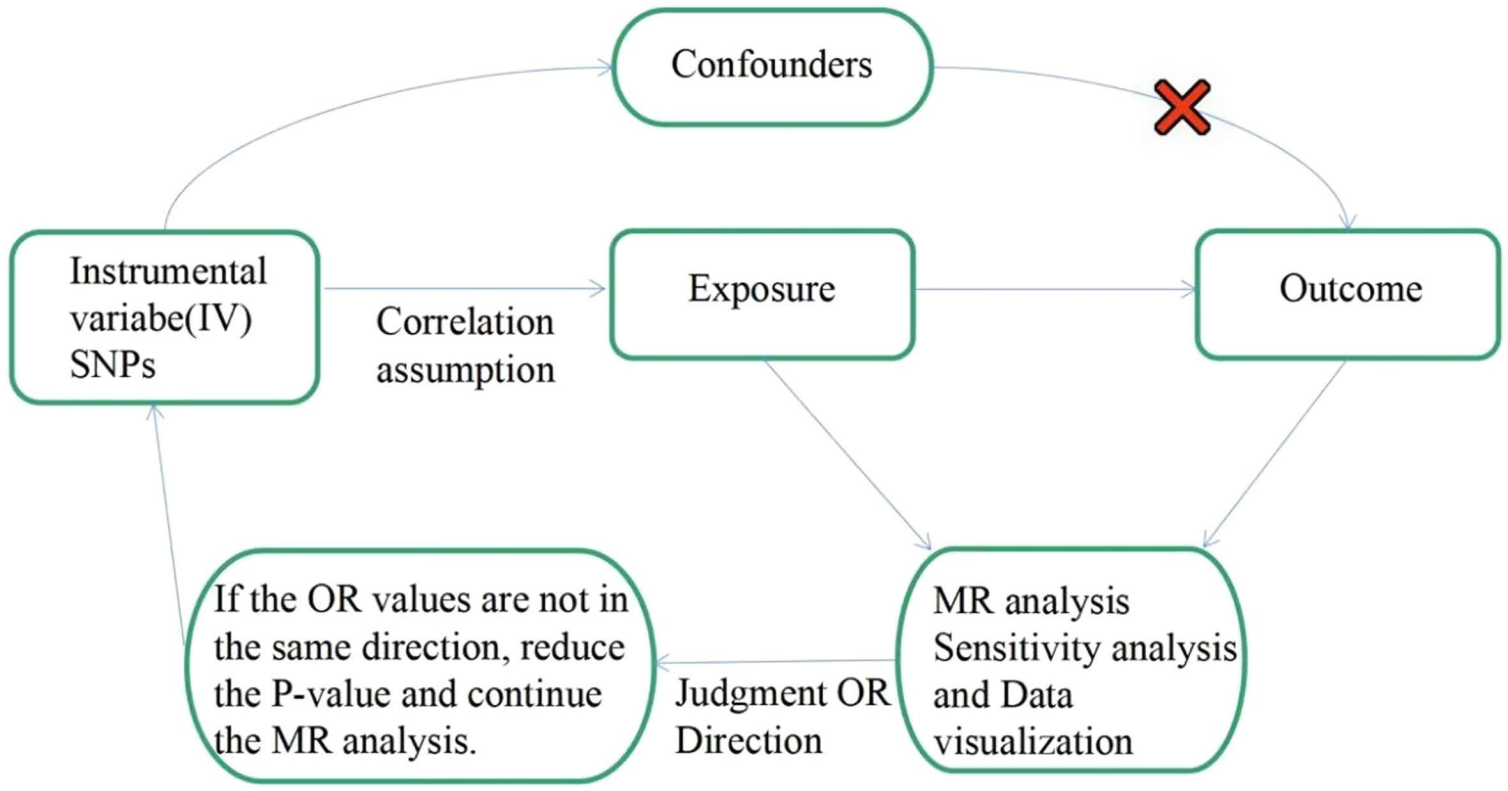
Flow chart: This is a schematic diagram of the bidirectional two-sample MR analysis of MDD with migraine subtypes (MA and MO). Three major assumptions of the MR analysis are as follows: Assumption 1: Genetic variation as an instrumental variable must be genuinely associated with exposure (MDD or migraine). Assumption 2: Exposure-outcome confounders have no effect on genetic variation. Assumption 3: Genetic variation affects outcome (migraine or MDD) through exposure (MDD or migraine) only, independent of other pathways. MR, Mendelian randomization; SNPs: single nucleotide polymorphisms; OR: odds ratio.

To investigate the genetic causality between MDD and migraine, we used a bidirectional MR study approach. To be a valuable tool for causal inference in MR studies, genetic variation must satisfy three fundamental criteria. Assumption 1: Genetic variation as an instrumental variable must be genuinely associated with exposure (MDD or migraine). Assumption 2: Exposure-outcome confounders have no effect on genetic variation. Assumption 3: Genetic variation affects outcome (migraine or MDD) through exposure (MDD or migraine) only, independent of other pathways. Figure 1 shows an overview of our study design. This MR study was conducted using a previously published, publicly available, large-scale GWAS abstract dataset. All participants provided written informed consent in the corresponding original GWAS.

### 2.2. Data source

#### 2.2.1. MDD data

The MDD and migraine data for our research were acquired from the IEU Open GWAS database, which is available *via* the GWAS catalog.^1^ The GWAS ID for the MDD we analyzed is ieu-b-102. Howard et al. conducted a meta-analysis of data on 807,553 individuals (246,363 cases and 561,190 controls) from the three most extensive genome-wide association studies of depression (23). The study incorporated many subtypes of depression. As a result, we only used MDD data generated from PGC data in that study (170,756 cases and 329,443 controls). It involved over 8 million SNPs and included both male and female European populations.

#### 2.2.2. Migraine data

Migraine is usually divided into migraine with aura and migraine without aura. Therefore, we analyzed migraine (8,547 cases and 176,107 controls), MA (3,541 cases and 176,107 controls), and MO (3,215 cases and 176,107 controls) from the publicly available GWAS database. The GWAS IDs for migraine, MA, and MO were finn-b-G6 MIGRAINE, finn-b-G6 MIGRAINE WITH AURA, and finn-b-G6 MIGRAINE NO AURA, respectively. All three data on migraine were derived from the same study involving more than 16 million SNPs, including male and female European populations.

### 2.3. Instrumental variable selection

The selected IVs should satisfy three basic assumptions of the MR analysis described previously. First, to obtain SNPs significantly associated with MDD, we set *p <* 5 × 10^−8^ as the genome-wide significance threshold. A relaxed threshold was used to acquire more IVs associated with the exposure of interest when obtaining IVs for migraine and subtypes, with the maximum threshold set to 5 × 10^−6^. Other investigations have reported on this threshold adjustment (24, 25). Meanwhile, since the presence of linkage disequilibrium (LD) would lead to biased results, in the final analysis, we set the LD of SNPs significantly associated with exposure should satisfy *r*^2^<0.001 and KB > 10,000. Our MR analysis excluded palindromic SNPs with intermediate allele frequencies. In addition, we quantified the strength of the genetic tool for all SNPs with an F-statistic calculated as β^2^/se^2^, and the F-statistic for IV as a follow-up analysis was higher than 10 (26).

### 2.4. Mendelian randomization analysis

Statistical analysis was performed using the R programming language (version 4.1.2). The MR analysis was based on the “TwoSampleMR” package (version 0.5.6), and the “MRPRESSO” package (version 1.0) was used to apply MRPRESSO analysis to identify outliers and detect pleiotropy.

For the causal analysis between exposure and outcome, we used random effects inverse variance weighting (IVW) as the primary analysis method, supplemented by MR-Egger, weighted median, simple mode, and weighted mode. Since the indicators used as outcomes were all dichotomous variables, we transformed the ratio estimates to obtain the corresponding dominance ratios (OR) and 95% confidence intervals (95% CI). When the OR values by conversion are not in the same direction, we decrease the *p*-value and continue with the MR analysis just performed. When the IVW method can provide more significant assistance in the study, it implies that all SNPs included in the analysis can be used as valid IVs (27). The pleiotropy of genetic variants may cause the three major assumptions for IVs to fail. The weighted median gives an accurate estimate based on the assumption that the number of valid IVs is 50%. At this point, the causal impact can still be computed accurately (28, 29). The MR-Egger regression technique implies that all IVs are invalid, and its estimation accuracy is relatively low (30, 31). The simple mode and weighted mode were also used to assess the robustness of the MR results (32).

### 2.5. Heterogeneity and horizontal pleiotropy

In addition, we will perform a series of sensitivity analyses, including heterogeneity and pleiotropy. IVW and MR-Egger regression were used to test for heterogeneity, and Q statistics were produced to quantify it (33). If there was heterogeneity, we conducted the study using IVW with random effects. Horizontal pleiotropy is essential for our study because being affected by horizontal pleiotropy may lead to unstable effect estimates. The MR-Egger intercept method calculates the intercept term available after the linear regression analysis to determine the likelihood of horizontal pleiotropy (34). The MR-PRESSO examination assesses the total pleiotropy of the study and examines for abnormal SNPs that may have horizontal pleiotropy (28). We utilized the software program to increase the number of distributions in the MR-PRESSO analysis to 5,000 and then performed a global test to notice whether there was pleiotropy in the study. The robustness of the MR analysis results was further evaluated by comparing the impacts before and after the removal of aberrant SNPs (35).

### 2.6. Data visualization

We used a leave-one-out analysis to examine the impact of individual SNPs on the causal correlation between MDD and migraine risk to prevent bias in the findings due to the pleiotropy of individual SNPs (36). The publication bias was evaluated by examining funnel plots for symmetry and assessing possible directional pleiotropy (37). Forest plots were used to examine effect estimates between genetic variants and MDD or migraine, and combined effects were calculated using the MR-Egger regression with IVW (28).

## 3. Results

A bidirectional, two-sample MR analysis was used to investigate the causative link between MDD levels and the risk of migraine and its subtypes. Our MR findings demonstrated a link between genetic vulnerability to MDD and an elevated risk of migraine and its subtypes; however, a link between migraine risk and MDD levels could not be established.

### 3.1. Causal effects of MDD on migraine and its subtypes

#### 3.1.1. Selection of instrumental variables

The publicly accessible MDD GWAS dataset was retrieved using the R programming language. We included 50 SNPs that were both substantially (*p* < 5E-08) linked with exposure (MDD) and independent (*r*^2^ < 0.001 and KB > 10,000). Some SNPs not detected in the result dataset were eliminated when utilizing these SNPs to correlate with the concluding GWAS dataset. One SNP was lost in the three MDD-migraine, MDD-MA, and MDD-MO analysis groups (rs35469634). After that, we removed two palindromic SNPs with intermediate allele frequencies from all three investigations (rs2876520 and rs4730387). It is worth noting that the OR direction of the MR-Egger transformation was inconsistent with other approaches when assessing MDD and MO; thus, we decreased P 10E-09 and repeated the MR study. Finally, 47 SNPs were identified as IVs in the MDD versus migraine and MA analysis (Supplementary Table 1), and 27 SNPs were identified as IVs in the MDD versus MO analysis (Supplementary Table S1). All F-statistics for the instrumental variables utilized in the final analysis were more extensive than 10 (MDD-Migraine and MDD-MA: mean value of 30–78, range of 39; MDD-MO: mean value of 34–78, range of 44). It was suggested that these are robust IVs and satisfy the strong correlation assumption of MR.

#### 3.1.2. Two-sample Mendelian randomization analysis

**Figure 2:**
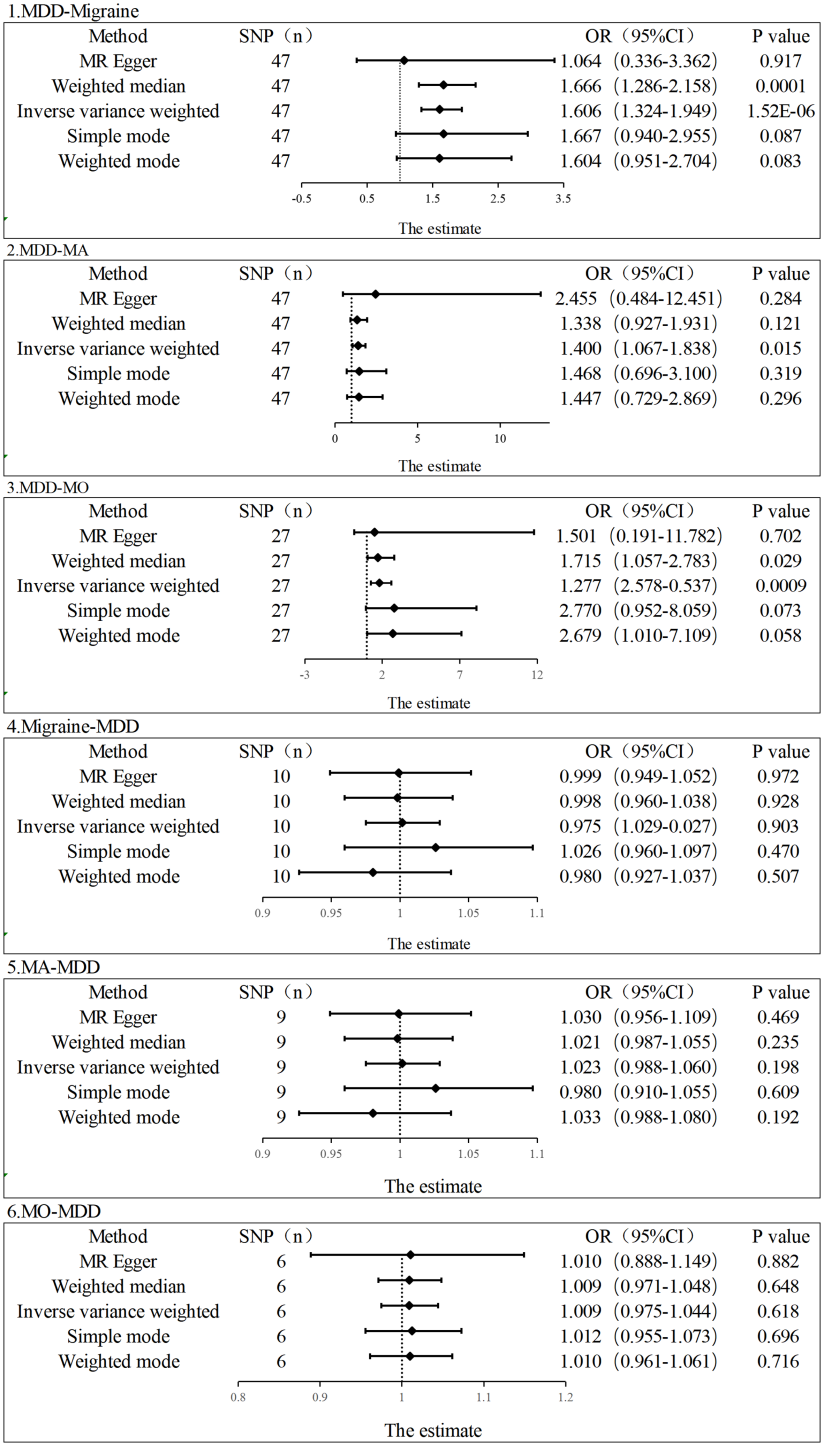
Estimation of the causal relationship between MDD and migraine (MA and MO) using different MR methods. An OR value greater than 1 suggests that the exposure indicator is a risk factor while the opposite is a protective factor.

**Figure 3:**
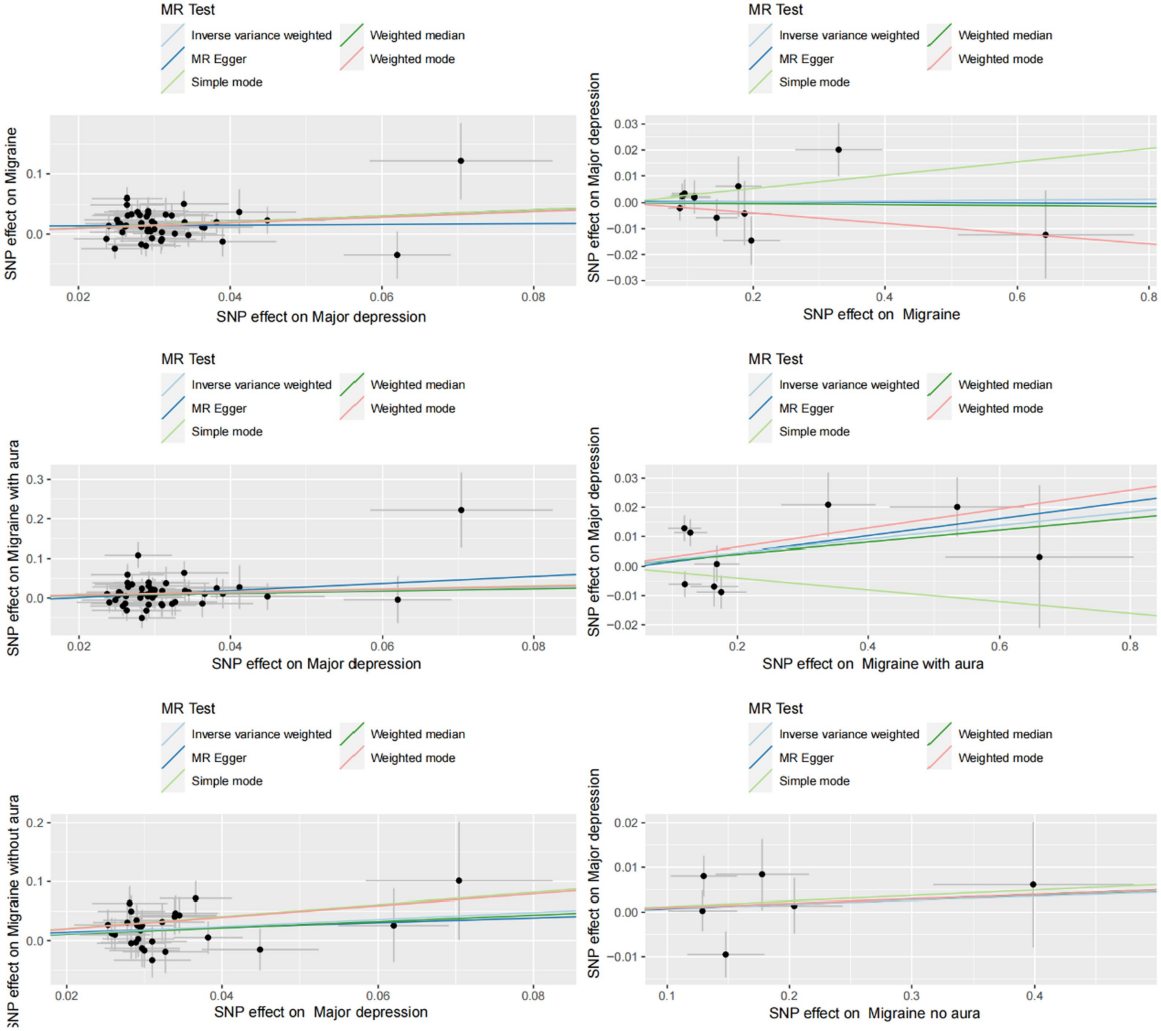
Scatter plot of genetic correlation between MDD and migraine (including MA and MO) by different MR analysis methods.

In the current study, the MR analysis had more than 80% statistical power. IVW was used as the primary method of analysis, which revealed a causal relationship between genetic susceptibility to MDD and increased risk of migraine and its subtypes (MDD-Migraine: OR, 1.606, 95% CI, 1.324–1.949, *p* = 1.52E-06; MDD-MA: OR, 1.400, 95%CI, 1.067–1.8378, *p* = 0.015; MDD-MO: OR, 1.814, 95%CI, 1.277–2.578, *p* = 0.0008). Secondary analysis methods included MR-Egger (MDD-Migraine: OR, 1.064, *p* = 0.917; MDD-MA: OR, 2.455, *p* = 0.284; MDD-MO: OR, 1.501, *p* = 0.702), weighted median (MDD-Migraine: OR, 1.666, *p* = 0.0001; MDD-MA: OR, 1.338, *p* = 0.121; MDD-MO: OR, 1.715, *p* = 0.029), weighted mode (MDD-Migraine: OR, 1.604, *p* = 0.083; MDD-MA: OR, 1.468, *p* = 0.319; MDD-MO: OR, 2.679, *p* = 0.058), and simple mode (MDD-Migraine: OR, 1.667, *p* = 0.087; MDD-MA: OR, 2.455, p = 0.284; MDD-MO: OR, 2.770, *p* = 0.073). The resulting OR values were all greater than 1 after transforming the relative risk ratios (Figures 2, 3).

#### 3.1.3. Sensitivity analysis and visualization

**Figure 4:**
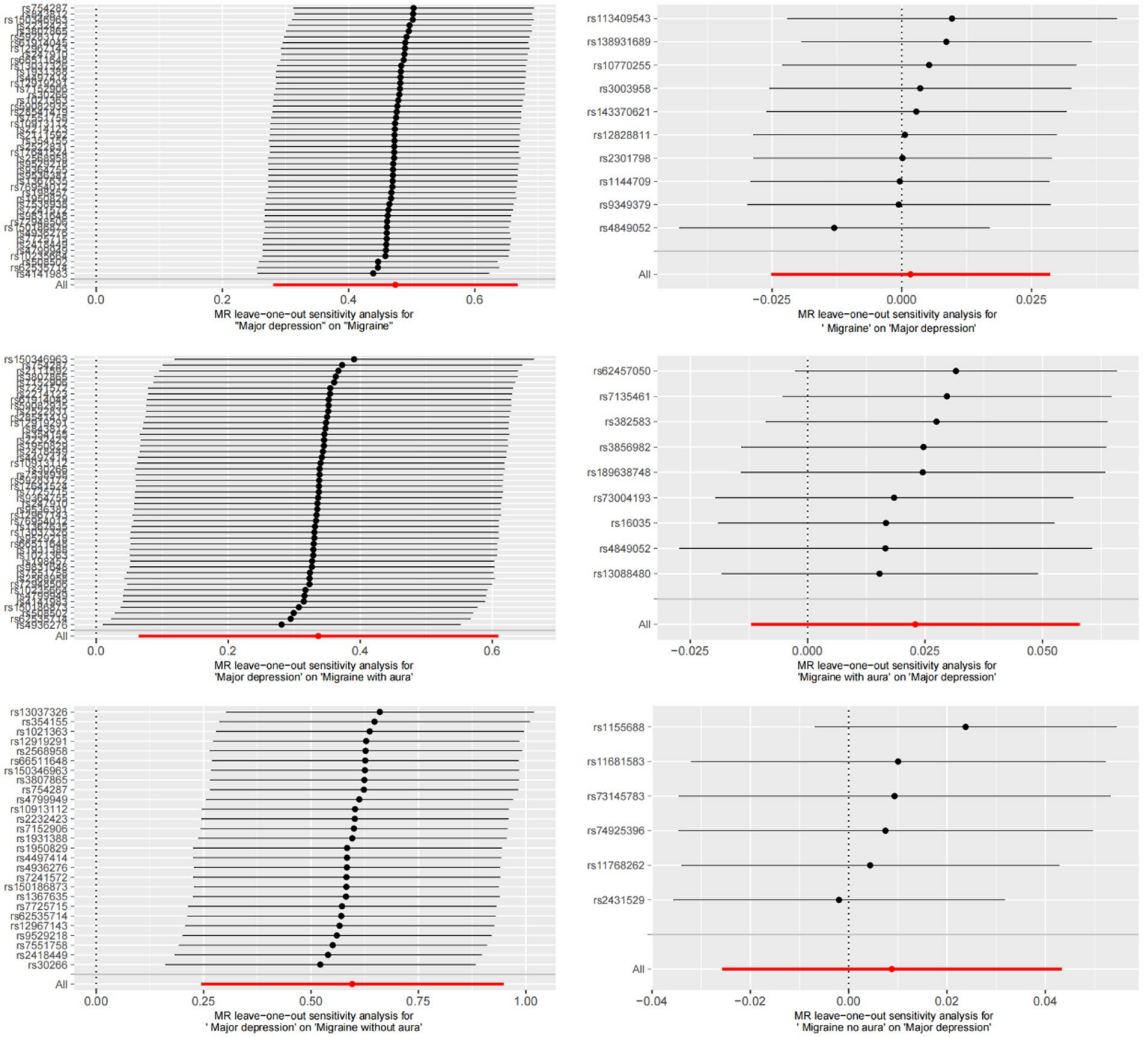
Bidirectional leave-one-out sensitivity analysis between MDD and migraine (including MA and MO). Red lines represent estimates from IVW tests. IVW: inverse variance weighted.

MR-Egger regression and IVW analysis were used to detect heterogeneity. MR-Egger regression (MDD-Migraine: Cochran’s *Q* = 52.376, *p* = 0.210; MDD-MA: Cochran’s *Q* = 46.704, *p* = 0.402; MDD-MO: Cochran’s *Q* = 21.435, *p* = 0.668) and IVW (MDD-Migraine: Cochran’s *Q* = 52.967, *p* = 0.223; MDD-MA: Cochran’s *Q* = 47.195, *p* = 0.423; MDD-MO: Cochran’s *Q* = 21.468, *p* = 0.717) indicated that there was no heterogeneity in the study (Supplementary Table S2). Funnel plots for the visualization of heterogeneity are shown in Supplementary Figure S1. The MR-Egger intercept did not show horizontal pleiotropy (MDD-Migraine: Egger intercept, 0.013, *p* = 0.480; MDD-MA: Egger intercept, −0.017, *p* = 0.495; MDD-MO: Egger intercept, 0.018, *p* = 0.480). The MR-PRESSO test found no outliers, and the global test showed no pleiotropy (global test: MDD-Migraine: *p* = 0.269; MDD-MA: *p* = 0.471; MDD-MO: *p* = 0.725) (Supplementary Table S2). We used the leave-one-out method to eliminate SNPs one at a time to determine whether the causal association was due to a single IV, and the final results demonstrated that the TSMR analysis results were robust (Figure 4). Forest plots for MR analyses of the relationship between MDD and migraine (both MA and MO) (Supplementary Figure S2).

### 3.2. Reverse TSMR analysis

In contrast, in TSMR, migraine (including MA and MO) was the exposure factor, and MDD was the outcome factor. To obtain more IVs, we set the value of *p* to less than 5 × 10–6. In addition, after the setting of chain imbalance (*r*^2^ < 0.001 and KB > 10,000), we ensured that the included IVs were following the core assumptions of MR and removing SNPs not present in the outcome dataset, and removing palindromic SNPs with intermediate allele frequencies. Finally, for the three exposure datasets of migraine, MA and MO, 10 SNPs, 9 SNPs, and 6 SNPs were included for MR analysis, respectively (Supplementary Table S1). The F-statistics were all greater than 10 (mean: 23, range: 21–31). The MR results did not support a relationship between genetic migraine susceptibility (including MA and MO) and an increased risk of MDD causality (IVW, Migraine-MDD: OR, 1.002, 95% CI, 0.975–1.029, *p* = 0.903; MA-MDD: OR, 1.023, 95%CI, 0.988–1.060, *p* = 0.198; MO-MDD: OR, 1.001, 95%CI, 0.975–1.044, *p* = 0.618). The heterogeneity test revealed that heterogeneity existed in the MA-MDD analysis (MR-Egger: Cochran’s Q, 23.399, *p* = 0.001; IVW: Cochran’s *Q* = 23.515, *p* = 0.003) but not in the Migraine-MDD and MO-MDD analyses (Migraine-MDD, MR-Egger: Cochran’s *Q* = 9.037, *p* = 0.339; IVW: Cochran’s *Q* = 9.054, *p* = 0.432; MO-MDD, MR-Egger: Cochran’s Q = 7.628, *p* = 0.106; IVW: Cochran’s Q, 7.629, *p* = 0.178). For the horizontal pleiotropy test, the MR-Egger intercept and the global MR-PRESSO test did not detect any abnormalities in the analysis between migraine and MO levels and MDD risk (Migraine-MDD: Egger intercept, 0.0004, *p* = 0.908; MDD-MO: Egger intercept, −0.0003, *p* = 0.980; global test: Migraine-MDD: *p* = 0.380; MO-MDD:*p* = 0.221). For the study of MA levels and MDD risk, the MR-Egger intercept indicated no pleiotropy (MA-MDD: Egger intercept, −0.00134, *p* = 0.857). However, the MR-PRESSO global test suggested the existence of some level of pleiotropy (global test: MA-MDD: *p* = 0.009).

## 4. Discussion

We used a bidirectional TSMR approach based on publicly available GWAS summary data to evaluate whether there is a bidirectional causal relationship between MDD and migraine (including migraine, MA, and MO). Our MR analysis revealed that MDD increased the probability of acquiring a migraine (migraine, MA, and MO). However, our results did not support a causal relationship between genetic susceptibility to migraine (migraine, MA, and MO) and increased risk of MDD. Except for the MA-MDD study, no outliers existed in the sensitivity analysis of the remaining comments, indicating the robustness of the findings.

Migraine is a common and debilitating neurological disorder that frequently co-occurs with sleeplessness, anxiety, depression, and epilepsy (38). Many studies have found a link between migraine and depression (39, 40). Migraine is a risk factor for depression, and the incidence and severity of migraine attacks rise during the depression and vice versa (41). Similarly, Jat et al. (42) discovered that 6.6% of patients with depression had MA, and 26.1% had MO in cross-sectional research. Kuan et al. (43) looked at the incidence of common disease combinations in 387,2,451 patients and found that depression was commonly associated with anxiety and migraine in all subgroups. Recent research indicates that the association between migraine and MDD is bidirectional. Furthermore, migraine and depression have significant genetic overlaps in terms of hereditary impact (44).

However, the causal relationship between migraine and depression remains unclear. The majority of earlier epidemiological research was case–control designs or cross-sectional studies with a hazy chronological order that failed to clarify causality. Furthermore, previous observational studies were plagued by insufficient sample numbers, difficulty in avoiding reverse causation, and confounding factors. Due to a more substantial study design, we were able to better show the causal link between exposure and outcome in the current study using a bidirectional TSMR analytic approach.

With the current study, we only found causal relationships between MDD and migraine (both MA and MO). Our study does not support reverse causality, most likely due to study constraints. This connection could be explained in several ways. One of the possible causes is hereditary factors. According to research, family members of depressed patients are more prone to suffer from migraines (45). Yang et al. found in an Australian study of 5,319 twin pairs that co-twins of probands reporting any depression had a significantly higher relative risk (RR) (RR = 1.18, 95% CI: 1.11–1.26) for any migraine than co-twins of controls (46). In addition, the role of serotonin in depression is well known, and reduced serotonin levels have been a focus of depression treatment (47). In a study of 186 depressed patients, Lee et al. (48) discovered that the serotonin transporter-linked polymorphic region (5HTTLPR) was positively associated with depression. Low serotonin levels, on the other hand, have been related to cortical spreading depression and migraine risk (49). Hence, we speculate that MDD may play a role in migraine through 5HT levels. Subsequently, we believe that structural alterations in the brain can have a significant impact. Some research has discovered aberrant brain activity in migraines and depression (50–52). Ma et al. (51) found that migraine and depression influence the left medial prefrontal cortex by evaluating four groups of migraine and depression patients. Furthermore, migraine patients with depression show unusual growth of the right thalamus and sphenoid in the pain and mood management brain areas. Yang et al. (50) used MRI to examine the right paracentral lobule and sphenoid functional changes in 93 patients with migraine and depression. We considered that MDD might increase the risk of migraine by altering brain activity.

Depression and migraine are frequently coexisting conditions that require long-term therapy. On the contrary, their interval increases medication resistance, interferes with treatment adherence, complicates diagnosis, has several social ramifications, and substantially impacts patients’ quality of life. This research shows a possible causal relationship between MDD levels and migraine risk, which may alert doctors to the importance of giving close attention to migraine symptoms in depressed patients. In clinical practice, routinely monitoring depressed patients for migraine-related signs may be helpful. It is also critical to pay close attention to the mixed symptoms of depression or migraine.

The advantages of our bidirectional TSMR study are as follows. First, we used the MR analysis method, SNPs with high association strength (*F* > 10) as instrumental variables, and the experimental design was similar to randomized controlled trials. Randomized controlled trials are commonly used in clinical practice and provide a high level of evidence, but they have drawbacks, including high costs and limited sample size. The MR study approach effectively avoids reversing causality and confounding factors. Second, the data we used were all from the GWAS database, which is all European population samples, effectively reducing the bias of population heterogeneity. Third, the results of our analysis may have implications for healthcare policy. Uncovering the causal relationship between MDD and migraine (MA and MO) may influence public health policies regarding prevention and treatment.

However, our study has several limitations. First, we acknowledged heterogeneity and horizontal pleiotropy in the study’s analysis of MA and MDD. The explanation for our findings could be due to a small sample size. Moreover, we used data on MDD not depressive symptoms, and the results obtained do not generalize to people with depressive symptoms that cannot be diagnosed as MDD. More studies on this subject should be carried out in the future. Second, we could not determine the association between the depression category and migraine intensity (low-frequency episodic migraine and high-frequency episodic migraine). Women had a higher prevalence of both MDD and migraine than men. However, because our data came from public databases, we could not undertake a factor-specific subgroup analysis such as age and gender. Third, the association between major depression and migraine is mediated by multiple factors and is not entirely determined by genetic factors. Although our study excluded confounding factors, we still could not completely avoid the interference of some factors, such as occupation, family, and other psychosocial factors, that might have influenced our study. MDD and migraine are chronic episodic disorders with more external confounding factors as they progress from mild to severe, which may have an effect on our study. Fourth, all of the subjects in the GWAS data were of European descent, and whether the results can be extrapolated to their populations requires further investigation. Fifth, increasing the GWAS sample size can enhance IV strength, and a larger-scale GWAS is necessary for in-depth research.

## 5. Conclusion

The bidirectional TSMR analysis indicated a relationship between MDD susceptibility and increased migraine risk (migraine, MA, and MO). On the other hand, the reverse investigation of causation was not supported.

## Data availability statement

The original contributions presented in the study are included in the article/Supplementary material, further inquiries can be directed to the corresponding author/s.

## Ethics statement

Ethical review and approval was not required for the study on human participants in accordance with the local legislation and institutional requirements. Written informed consent from the patients/participants or patients/participants’ legal guardian/next of kin was not required to participate in this study in accordance with the national legislation and the institutional requirements.

## Author contributions

XL, BX, XT, and SL: study design, data collection, and statistical analysis. J-HQ, JG, and JL: supervision. XL, BX, and XT: writing – original draft. XL, BX, XT, J-HQ, JG, and JL: writing – review and editing. All authors contributed to the article and approved the submitted version.

## Funding

This study was funded by research on the Academic Viewpoints, Unique Diagnostic and Treatment Methods, and Major Diseases Prevention and Treatment Experience of Illustrious Senior Traditional Chinese Medicine Practitioners in Western China (no. 2018YFC1704104) and Sichuan Administration of Traditional Chinese Medicine (no. 2021MS412 and no. CKY2022090).

## Conflict of interest

The authors declare that the research was conducted in the absence of any commercial or financial relationships that could be construed as a potential conflict of interest.

## Publisher’s note

All claims expressed in this article are solely those of the authors and do not necessarily represent those of their affiliated organizations, or those of the publisher, the editors and the reviewers. Any product that may be evaluated in this article, or claim that may be made by its manufacturer, is not guaranteed or endorsed by the publisher.

## Footnotes

^1^ https://www.ebi.ac.uk/gwas
