# Supplementary material for: The relationship between major depression and migraine: A bidirectional two-sample Mendelian randomization study
Source: Front Neurol. 2023 Apr 14;14:1143060. doi: 10.3389/fneur.2023.1143060 (PMC10140565; doi:10.3389/fneur.2023.1143060)
Supplement: Supplementary file 1 [file Data_Sheet_1.zip › Supplementary Figure 1.docx]

Supplementary Figure 1:

Funnel plot


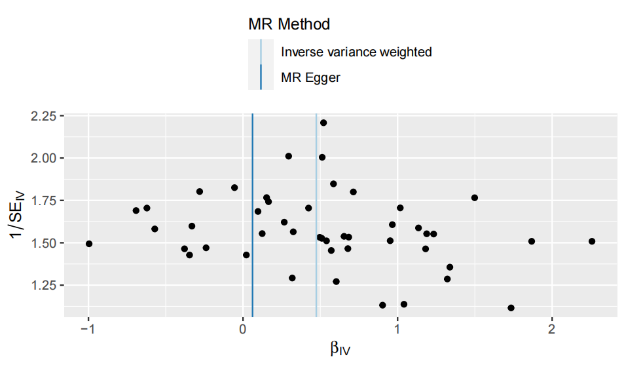

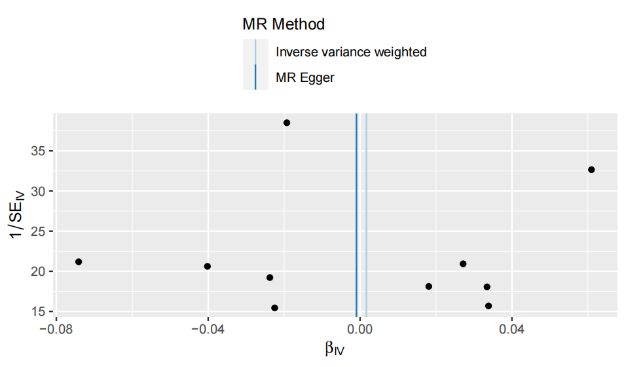


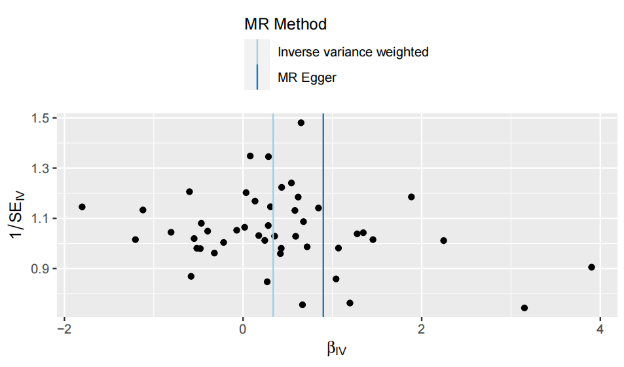

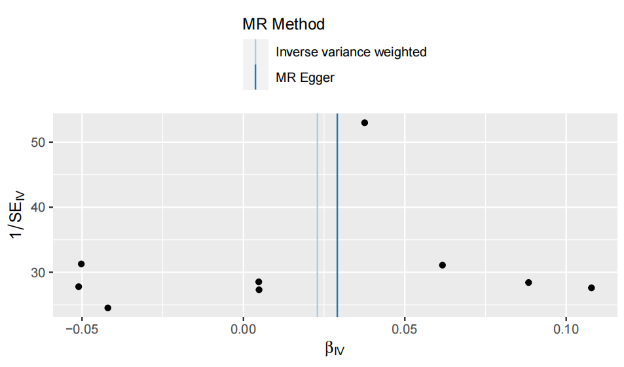


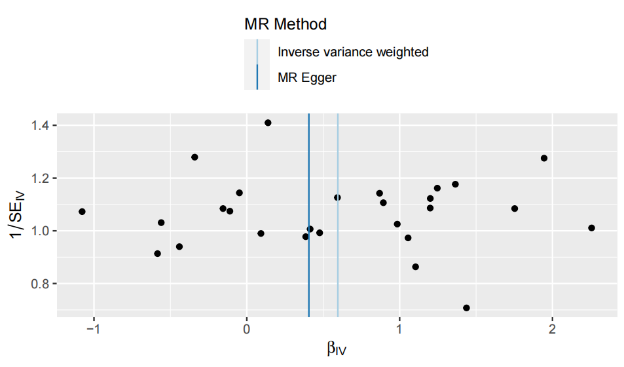

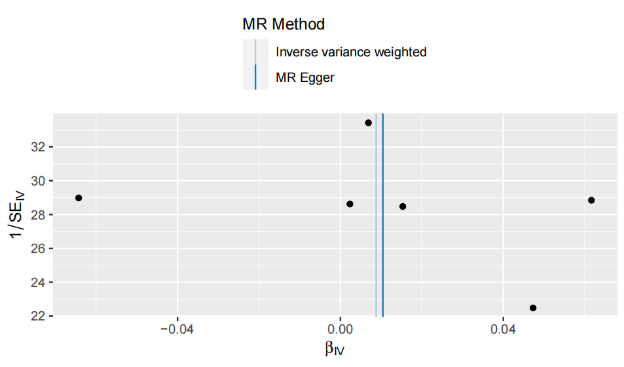


Supplementary Figure 1: Funnel plot of SNPs. On the left, from top to bottom, is the funnel plot of the analysis between MDD and Migraine, MA, and MO. On the right is the funnel plot of the analysis between Migraine, MA, MO and MDD.
