# Supplementary material for: The relationship between major depression and migraine: A bidirectional two-sample Mendelian randomization study
Source: Front Neurol. 2023 Apr 14;14:1143060. doi: 10.3389/fneur.2023.1143060 (PMC10140565; doi:10.3389/fneur.2023.1143060)
Supplement: Supplementary file 1 [file Data_Sheet_1.zip › Supplementary Figure 2.docx]

Supplementary Figure 2:


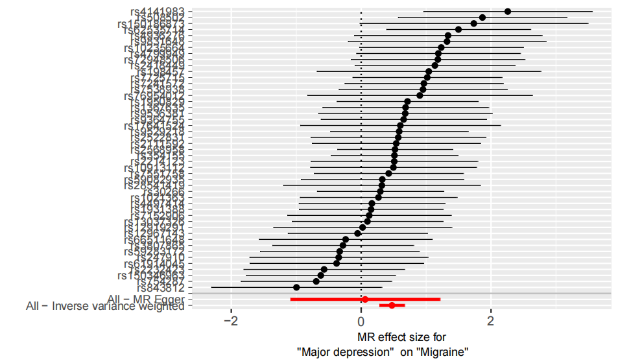

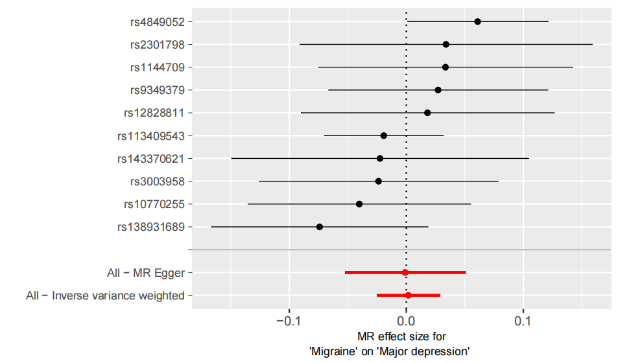

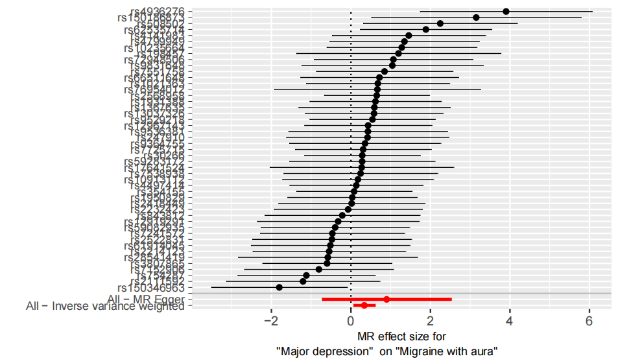

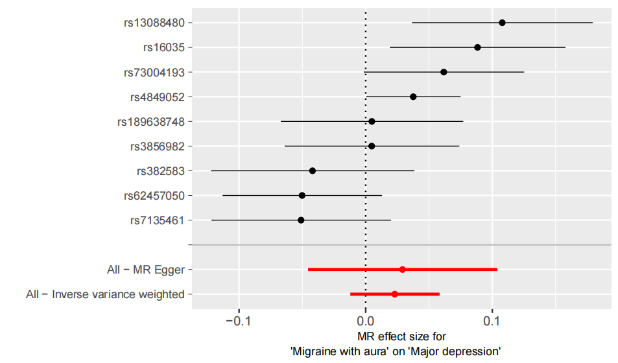

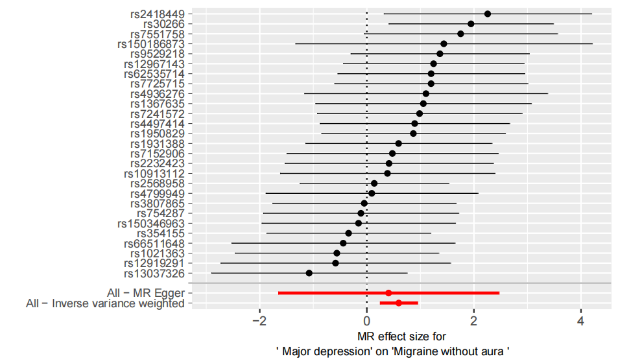

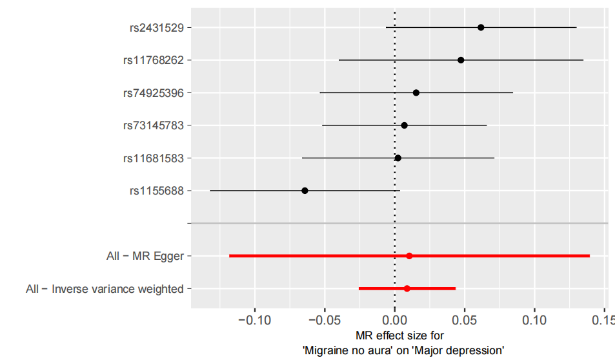


Supplementary Figure 2: Forest plot of the bidirectional causal effect of MDD with migraine (both MA and MO) and associated SNPs. The red and black dots/lines indicate the causal estimates of the bidirectional risk between MDD and migraine (both MA and MO).
