# Supplementary material for: The relationship between major depression and migraine: A bidirectional two-sample Mendelian randomization study
Source: Front Neurol. 2023 Apr 14;14:1143060. doi: 10.3389/fneur.2023.1143060 (PMC10140565; doi:10.3389/fneur.2023.1143060)
Supplement: Supplementary file 1 [file Data_Sheet_1.zip › Supplementary Table 1.docx]

Supplementary Materials Table 1:

Instrumental Variable

| 1.MDD-Migraine and MDD-MA | | | | | | | |
| --- | --- | --- | --- | --- | --- | --- | --- |
| SNP | Chr | EA | OA | Beta | Eaf | SE | Pval |
| rs10913112 | 1 | T | C | -0.0262 | 0.378 | 0.0045 | 4.53E-09 |
| rs17641524 | 1 | T | C | -0.03 | 0.2101 | 0.0053 | 1.50E-08 |
| rs2568958 | 1 | A | G | 0.0382 | 0.6042 | 0.0044 | 2.90E-18 |
| rs354155 | 1 | C | G | -0.0449 | 0.0923 | 0.0075 | 1.75E-09 |
| rs4141983 | 1 | C | T | -0.0264 | 0.326 | 0.0046 | 9.69E-09 |
| rs7538938 | 1 | C | T | 0.0251 | 0.5599 | 0.0043 | 7.29E-09 |
| rs7551758 | 1 | G | T | 0.0283 | 0.5329 | 0.0043 | 5.11E-11 |
| rs2111592 | 2 | A | G | 0.0263 | 0.3141 | 0.0046 | 1.35E-08 |
| rs72948506 | 2 | A | G | 0.0265 | 0.2975 | 0.0047 | 1.71E-08 |
| rs66511648 | 3 | C | T | 0.0297 | 0.284 | 0.0048 | 6.03E-10 |
| rs76954012 | 3 | A | T | 0.0412 | 0.0931 | 0.0074 | 2.41E-08 |
| rs843812 | 3 | A | G | 0.0248 | 0.4117 | 0.0044 | 1.41E-08 |
| rs9831648 | 3 | T | G | -0.0292 | 0.7739 | 0.0052 | 1.59E-08 |
| rs247910 | 5 | G | A | 0.0237 | 0.457 | 0.0043 | 4.71E-08 |
| rs30266 | 5 | A | G | 0.0366 | 0.3271 | 0.0046 | 1.43E-15 |
| rs7725715 | 5 | A | G | 0.029 | 0.5343 | 0.0043 | 1.61E-11 |
| rs150186873 | 6 | C | A | 0.0704 | 0.0327 | 0.012 | 4.51E-09 |
| rs2214123 | 6 | G | A | -0.0261 | 0.6466 | 0.0045 | 8.56E-09 |
| rs2232423 | 6 | G | A | -0.062 | 0.1056 | 0.007 | 1.14E-18 |
| rs9364755 | 6 | G | A | 0.0283 | 0.2262 | 0.0051 | 3.49E-08 |
| rs10235664 | 7 | C | T | -0.027 | 0.2529 | 0.0049 | 4.68E-08 |
| rs150346963 | 7 | T | C | 0.0283 | 0.4118 | 0.0044 | 1.16E-10 |
| rs2522831 | 7 | C | T | 0.024 | 0.4739 | 0.0043 | 2.11E-08 |
| rs3807865 | 7 | A | G | 0.031 | 0.4105 | 0.0044 | 1.09E-12 |
| rs59082935 | 7 | T | C | 0.0363 | 0.1342 | 0.0066 | 3.07E-08 |
| rs1931388 | 9 | G | A | -0.0295 | 0.4042 | 0.0044 | 1.68E-11 |
| rs2418449 | 9 | C | T | -0.0281 | 0.281 | 0.0048 | 4.25E-09 |
| rs59283172 | 9 | A | G | -0.039 | 0.1081 | 0.007 | 2.41E-08 |
| rs62535714 | 9 | A | G | 0.0339 | 0.1639 | 0.0058 | 4.69E-09 |
| rs1021363 | 10 | G | A | -0.03 | 0.6434 | 0.0045 | 2.29E-11 |
| rs198457 | 11 | T | C | -0.0315 | 0.1886 | 0.0056 | 1.90E-08 |
| rs4497414 | 11 | C | T | 0.0291 | 0.44 | 0.0044 | 2.93E-11 |
| rs4936276 | 11 | C | G | 0.0278 | 0.622 | 0.0044 | 3.57E-10 |
| rs61914045 | 12 | A | G | 0.0309 | 0.2034 | 0.0054 | 7.96E-09 |
| rs508502 | 13 | T | C | -0.0264 | 0.2992 | 0.0048 | 3.56E-08 |
| rs9529218 | 13 | T | C | -0.034 | 0.2031 | 0.0054 | 2.23E-10 |
| rs9536381 | 13 | T | C | 0.0255 | 0.3259 | 0.0046 | 2.62E-08 |
| rs1950829 | 14 | G | A | -0.0297 | 0.5173 | 0.0043 | 4.74E-12 |
| rs7152906 | 14 | C | T | 0.0258 | 0.5196 | 0.0043 | 1.87E-09 |
| rs754287 | 14 | A | T | -0.0289 | 0.3664 | 0.0045 | 1.31E-10 |
| rs28541419 | 15 | G | C | -0.0292 | 0.2308 | 0.0052 | 1.76E-08 |
| rs12919291 | 16 | C | G | 0.0327 | 0.1884 | 0.0055 | 3.09E-09 |
| rs12967143 | 18 | C | G | -0.0345 | 0.7012 | 0.0047 | 2.53E-13 |
| rs1367635 | 18 | C | T | 0.0253 | 0.5148 | 0.0043 | 4.35E-09 |
| rs4799949 | 18 | T | C | -0.0292 | 0.6684 | 0.0046 | 1.40E-10 |
| rs7241572 | 18 | A | G | 0.0323 | 0.2047 | 0.0054 | 2.43E-09 |
| rs13037326 | 20 | T | C | 0.031 | 0.2597 | 0.0049 | 2.40E-10 |
|  |  |  |  |  |  |  |  |
| 2.MDD-MO |  |  |  |  |  |  |  |
| SNP | Chr | EA | OA | Beta | Eaf | SE | Pval |
| rs10913112 | 1 | T | C | -0.0262 | 0.378 | 0.0045 | 4.53E-09 |
| rs2568958 | 1 | A | G | 0.0382 | 0.6042 | 0.0044 | 2.90E-18 |
| rs354155 | 1 | C | G | -0.0449 | 0.0923 | 0.0075 | 1.75E-09 |
| rs7551758 | 1 | G | T | 0.0283 | 0.5329 | 0.0043 | 5.11E-11 |
| rs66511648 | 3 | C | T | 0.0297 | 0.284 | 0.0048 | 6.03E-10 |
| rs30266 | 5 | A | G | 0.0366 | 0.3271 | 0.0046 | 1.43E-15 |
| rs7725715 | 5 | A | G | 0.029 | 0.5343 | 0.0043 | 1.61E-11 |
| rs150186873 | 6 | C | A | 0.0704 | 0.0327 | 0.012 | 4.51E-09 |
| rs2232423 | 6 | G | A | -0.062 | 0.1056 | 0.007 | 1.14E-18 |
| rs150346963 | 7 | T | C | 0.0283 | 0.4118 | 0.0044 | 1.16E-10 |
| rs3807865 | 7 | A | G | 0.031 | 0.4105 | 0.0044 | 1.09E-12 |
| rs1931388 | 9 | G | A | -0.0295 | 0.4042 | 0.0044 | 1.68E-11 |
| rs2418449 | 9 | C | T | -0.0281 | 0.281 | 0.0048 | 4.25E-09 |
| rs62535714 | 9 | A | G | 0.0339 | 0.1639 | 0.0058 | 4.69E-09 |
| rs1021363 | 10 | G | A | -0.03 | 0.6434 | 0.0045 | 2.29E-11 |
| rs4497414 | 11 | C | T | 0.0291 | 0.44 | 0.0044 | 2.93E-11 |
| rs4936276 | 11 | C | G | 0.0278 | 0.622 | 0.0044 | 3.57E-10 |
| rs9529218 | 13 | T | C | -0.034 | 0.2031 | 0.0054 | 2.23E-10 |
| rs1950829 | 14 | G | A | -0.0297 | 0.5173 | 0.0043 | 4.74E-12 |
| rs7152906 | 14 | C | T | 0.0258 | 0.5196 | 0.0043 | 1.87E-09 |
| rs754287 | 14 | A | T | -0.0289 | 0.3664 | 0.0045 | 1.31E-10 |
| rs12919291 | 16 | C | G | 0.0327 | 0.1884 | 0.0055 | 3.09E-09 |
| rs12967143 | 18 | C | G | -0.0345 | 0.7012 | 0.0047 | 2.53E-13 |
| rs1367635 | 18 | C | T | 0.0253 | 0.5148 | 0.0043 | 4.35E-09 |
| rs4799949 | 18 | T | C | -0.0292 | 0.6684 | 0.0046 | 1.40E-10 |
| rs7241572 | 18 | A | G | 0.0323 | 0.2047 | 0.0054 | 2.43E-09 |
| rs13037326 | 20 | T | C | 0.031 | 0.2597 | 0.0049 | 2.40E-10 |
|  |  |  |  |  |  |  |  |
| 3.Migraine-MDD | |  |  |  |  |  |  |
| SNP | Chr | EA | OA | Beta | Eaf | SE | Pval |
| rs138931689 | 1 | A | G | 0.197 | 0.04063 | 0.0431 | 4.86E-06 |
| rs4849052 | 2 | C | T | -0.3298 | 0.9834 | 0.066 | 5.91E-07 |
| rs1144709 | 6 | T | C | -0.0957 | 0.2502 | 0.0193 | 6.70E-07 |
| rs143370621 | 6 | G | A | -0.1868 | 0.04631 | 0.0396 | 2.32E-06 |
| rs9349379 | 6 | G | A | -0.0921 | 0.4511 | 0.0166 | 2.96E-08 |
| rs3003958 | 10 | A | G | 0.0884 | 0.3302 | 0.0176 | 5.07E-07 |
| rs10770255 | 12 | T | A | 0.1443 | 0.07509 | 0.0314 | 4.15E-06 |
| rs113409543 | 12 | G | C | -0.6429 | 0.004584 | 0.1331 | 1.36E-06 |
| rs12828811 | 12 | G | A | -0.1105 | 0.1452 | 0.0237 | 3.09E-06 |
| rs2301798 | 19 | T | C | -0.1773 | 0.06235 | 0.0346 | 3.08E-07 |
|  |  |  |  |  |  |  |  |
| 4.MA-MDD |  |  |  |  |  |  |  |
| SNP | Chr | EA | OA | Beta | Eaf | SE | Pval |
| rs4849052 | 2 | C | T | -0.5351 | 0.9835 | 0.1018 | 1.49E-07 |
| rs13088480 | 3 | C | T | 0.1187 | 0.4693 | 0.0247 | 1.58E-06 |
| rs189638748 | 4 | A | G | 0.6609 | 0.009006 | 0.1436 | 4.19E-06 |
| rs3856982 | 4 | C | T | -0.1683 | 0.1571 | 0.0339 | 7.02E-07 |
| rs73004193 | 6 | G | A | 0.3389 | 0.03346 | 0.0717 | 2.29E-06 |
| rs62457050 | 7 | T | C | 0.1752 | 0.132 | 0.0376 | 3.10E-06 |
| rs7135461 | 12 | A | C | 0.1195 | 0.5373 | 0.0247 | 1.36E-06 |
| rs16035 | 19 | G | T | -0.1279 | 0.5029 | 0.0248 | 2.45E-07 |
| rs382583 | 21 | C | T | 0.1643 | 0.8607 | 0.036 | 4.87E-06 |
|  |  |  |  |  |  |  |  |
| 5.MO-MDD |  |  |  |  |  |  |  |
| SNP | Chr | EA | OA | Beta | Eaf | SE | Pval |
| rs11681583 | 2 | G | A | 0.1288 | 0.3139 | 0.028 | 4.35E-06 |
| rs2431529 | 5 | G | A | -0.1298 | 0.382 | 0.0268 | 1.28E-06 |
| rs11768262 | 7 | A | C | -0.1776 | 0.1345 | 0.0379 | 2.85E-06 |
| rs1155688 | 12 | A | G | -0.1478 | 0.7745 | 0.0312 | 2.10E-06 |
| rs73145783 | 20 | G | A | 0.2039 | 0.1259 | 0.0392 | 2.03E-07 |
| rs74925396 | 22 | G | A | 0.3987 | 0.02799 | 0.0818 | 1.10E-06 |

Supplementary Materials Table 1: SNP: single nucleotide polymorphism; EA: effect allele; OA: non-effect allele; Chr: chromosome; EAF: effect allele frequency; Beta was obtained by allele-related effects; SE: standard error. Beta, SE, and Pval are SNP summary statistics; MDD: Major Depressive Disorder; MA: Migraine with aura; MO: Migraine without aura.
