# Supplementary material for: The relationship between major depression and migraine: A bidirectional two-sample Mendelian randomization study
Source: Front Neurol. 2023 Apr 14;14:1143060. doi: 10.3389/fneur.2023.1143060 (PMC10140565; doi:10.3389/fneur.2023.1143060)
Supplement: Supplementary file 1 [file Data_Sheet_1.zip › Supplementary Table 2.docx]

Supplementary Table 2:

Sensitivity Analysis

Heterogeneity test

| Exposure | Outcome | Heterogeneity test (MR-Egger) | | | Heterogeneity test (IVW) | | |
| --- | --- | --- | --- | --- | --- | --- | --- |
|  |  | Cochran's Q | Q_df | P | Cochran's Q | Q_df | P |
| MDD | Migraine | 52.376 | 45 | 0.210 | 52.967 | 46 | 0.223 |
| MDD | MA | 46.704 | 45 | 0.402 | 47.195 | 46 | 0.423 |
| MDD | MO | 21.435 | 25 | 0.668 | 21.468 | 26 | 0.717 |
| Migraine | MDD | 9.037 | 8 | 0.339 | 9.054 | 9 | 0.432 |
| MA | MDD | 23.399 | 7 | 0.001 | 23.515 | 8 | 0.003 |
| MO | MDD | 7.628 | 4 | 0.106 | 7.629 | 5 | 0.178 |

Horizontal pleiotropy test

| Exposure | Outcome | Horizontal pleiotropy test (MR-Egger) | | Horizontal pleiotropy test (MR-PRESSO) |
| --- | --- | --- | --- | --- |
|  |  | Intercept | P | Globle test Pval |
| MDD | Migraine | 0.013 | 0.480 | 0.269 |
| MDD | MA | -0.017 | 0.495 | 0.471 |
| MDD | MO | 0.013 | 0.480 | 0.725 |
| Migraine | MDD | 0.0005 | 0.908 | 0.380 |
| MA | MDD | -0.001 | 0.857 | 0.009 |
| MO | MDD | -0.0003 | 0.980 | 0.221 |

Supplementary Table 2: Sensitivity analysis of MR, including pleiotropy analysis and heterogeneity analysis.
